# Ovarian senescence increases liver fibrosis in humans and zebrafish with steatosis

**DOI:** 10.1242/dmm.019950

**Published:** 2015-09-01

**Authors:** Elena Turola, Salvatore Petta, Ester Vanni, Fabiola Milosa, Luca Valenti, Rosina Critelli, Luca Miele, Livia Maccio, Vincenza Calvaruso, Anna L. Fracanzani, Marcello Bianchini, Nazarena Raos, Elisabetta Bugianesi, Serena Mercorella, Marisa Di Giovanni, Antonio Craxì, Silvia Fargion, Antonio Grieco, Calogero Cammà, Franco Cotelli, Erica Villa

**Affiliations:** 1 Gastroenterology Unit, Department of Internal Medicine, University of Modena and Reggio Emilia, 41124 Modena, Italy; 2 Division of Gastroenterology, DiBiMIS, University of Palermo, 90128 Palermo, Italy; 3 Division of Gastroenterology and Hepatology, Department of Medical Sciences, University of Torino, 10126 Torino, Italy; 4 Department of Pathophysiology and Transplantation, Section Internal Medicine, Fondazione Ca’ Granda IRCCS Ospedale Maggiore Policlinico, 20122 Milano, Italy; 5 Institute of Internal Medicine, School of Medicine, Catholic University of the Sacred Heart, 00168 Rome, Italy; 6 Department of Pathology, University of Modena and Reggio Emilia, 41124 Modena, Italy; 7 Department of Biosciences, University of Milan, 20122 Milan, Italy

**Keywords:** Fibrosis, Menopause, Non-alcoholic fatty liver disease, Ovarian senescence, Zebrafish

## Abstract

Contrasting data exist on the effect of gender and menopause on the susceptibility, development and liver damage progression in non-alcoholic fatty liver disease (NAFLD). Our aim was to assess whether menopause is associated with the severity of liver fibrosis in individuals with NAFLD and to explore the issue of ovarian senescence in experimental liver steatosis in zebrafish. In 244 females and age-matched males with biopsy-proven NAFLD, we assessed anthropometric, biochemical and metabolic features, including menopausal status (self-reported); liver biopsy was scored according to ‘The Pathology Committee of the NASH Clinical Research Network’. Young and old male and female zebrafish were fed for 24 weeks with a high-calorie diet. Weekly body mass index (BMI), histopathological examination and quantitative real-time PCR analysis on genes involved in lipid metabolism, inflammation and fibrosis were performed. In the entire cohort, at multivariate logistic regression, male gender [odds ratio (OR): 1.408, 95% confidence interval (95% CI): 0.779-2.542, *P*=0.25] vs women at reproductive age was not associated with F2-F4 fibrosis, whereas a trend was observed for menopause (OR: 1.752, 95% CI: 0.956-3.208, *P*=0.06). In women, menopause (OR: 2.717, 95% CI: 1.020-7.237, *P*=0.04) was independently associated with F2-F4 fibrosis. Similarly, in overfed zebrafish, old female fish with failing ovarian function [as demonstrated by extremely low circulating estradiol levels (1.4±0.1 pg/µl) and prevailing presence of atretic follicles in the ovaries] developed massive steatosis and substantial fibrosis (comparable with that occurring in males), whereas young female fish developed less steatosis and were totally protected from the development of fibrosis. Ovarian senescence significantly increases the risk of fibrosis severity both in humans with NAFLD and in zebrafish with experimental steatosis.

## Abstract (precis)

**Summary:** This study provides clinical and experimental evidence for the different roles played by excess calorie intake in the development of NAFLD and fibrosis; these diseases are dependent on age and reproductive status.

## INTRODUCTION

Non-alcoholic fatty liver disease (NAFLD) affects about 20-30% of the general population (Bedogni et al., 2005) and is a leading cause of chronic liver disease worldwide (Marchesini et al., 2003; Petta et al., 2009). A relevant proportion of individuals with NAFLD is at risk of progression to cirrhosis and its complications (Petta and Craxi, 2010; Petta et al., 2009), and there is a high rate of cancer and cardiovascular events (Targher et al., 2008) in such individuals compared with subjects without fatty liver.

In NAFLD individuals, a number of factors like obesity, insulin resistance (IR) and necroinflammation (Argo et al., 2009; Bugianesi et al., 2006; Petta et al., 2011b; van der Poorten et al., 2008) have been reported to be involved in determining the severity of liver fibrosis and its progression. In addition, new players in the pathogenesis of liver damage in NAFLD are emerging, such as genetic background (Dongiovanni et al., 2013), fructose consumption (Abdelmalek et al., 2010) and hyperuricemia (Petta et al., 2011a).

Different lines of evidence indicate a higher risk of metabolic disturbances among menopausal females (Lovejoy et al., 2008), and accordingly, experimental studies show a protective anti-fibrogenic and anti-steatogenic effect of estrogens (Chow et al., 2011; Hewitt et al., 2004; Itagaki et al., 2005; Yasuda et al., 1999). Despite these interesting data, only a few studies have investigated the impact of gender and, in particular, of menopausal status on the severity of liver disease in individuals with NAFLD. Browning et al. (2004) report a higher prevalence of NAFLD in males with respect to females, this effect being restricted to the Caucasian population only, but not to Hispanic and African Americans. By contrast, some epidemiological studies from Germany (Volzke et al., 2007), the USA (Clark et al., 2002), Korea (Park et al., 2006) and Japan (Hamaguchi et al., 2012) show that menopause increases the prevalence and incidence of NAFLD. Similarly, other work suggests that hormone replacement therapy (HRT) is protective against NAFLD after menopause (McKenzie et al., 2006). Finally, a recent study from the USA has identified an independent risk factor for fibrosis severity in self-reported or age-presumed menopause among a cohort of males and females with non-alcoholic steatohepatitis (NASH) (Yang et al., 2014).

As an experimental model to test the relationship between steatosis and the development of fibrosis from a gender perspective, we chose zebrafish for its remarkable similarities with mammals in lipid metabolism. As in humans, dietary lipids are delivered from the intestine to the liver and from the liver to extra-hepatic tissues (Asaoka et al., 2013; Holtta-Vuori et al., 2010). Recently, Oka et al. (2010) have described a model of dietary-induced obesity in zebrafish, which shares common pathophysiological features with mammalian obesity.

**TRANSLATIONAL IMPACT**

**Clinical issue**

Non-alcoholic fatty liver disease (NAFLD) is the most common chronic liver disease in western countries with a prevalence of 20-30% in the overall population. It is characterised by a wide spectrum of clinical manifestations, from simple steatosis to steatohepatitis (NASH), to advanced fibrosis and cirrhosis, as well as by complications like decompensation, variceal bleeding and hepatocellular carcinoma (HCC). Different studies have demonstrated that non-alcoholic hepatic steatosis is less common in women than in men, that anti-estrogenic therapy increases the risk of non-alcoholic steatohepatitis and that the prevalence of NAFLD is lower in women of reproductive age. The aim of this study was to assess whether gender, age and menopause affect the susceptibility to liver damage both in humans with NAFLD and in zebrafish with experimental steatosis.

**Results**

In this study, the authors demonstrated that menopausal women have a higher prevalence of metabolic disturbances [including higher total cholesterol, higher homeostasis model assessment (HOMA) index – a measure of insulin resistance, type 2 diabetes and arterial hypertension] than women of reproductive age and that menopausal status increases the risk of having stage F2-F4 hepatic fibrosis (defined as: F2 moderate fibrosis, F3 severe fibrosis and F4 cirrhosis). The experimental data obtained in the model of diet-induced obesity in zebrafish are extremely similar to those found in humans, and include different susceptibilities to liver fibrosis according to reproductive phases. Histopathological examination of zebrafish liver showed that young female zebrafish are protected from the development of fibrosis, whereas all the other groups (young and old male fish, and old female fish) are not, thus reinforcing the hypothesis of a protective effect of estrogens and of a role for ovarian senescence in the development of hepatic fibrosis. Expression analysis of genes in zebrafish liver that are involved in lipid metabolism, inflammation and fibrosis showed a pattern reflecting that which leads to steatosis and fibrosis in mice and humans, and supports the hypothesis that simple overfeeding is able to induce liver steatosis and fibrosis.

**Implication and future directions**

The data reported in this study call for attention to the reproductive status of individuals with NAFDL and/or NASH, and suggests that this aspect should not be overlooked in the management of such individuals. Zebrafish data indicate that the model of diet-induced obesity, replicating features of obesity and steatosis in humans, could be very useful to study the long-term effects of excessive calorie intake on the development of steatosis and fibrosis from a gender and age perspective. The ease of the overfeeding protocol, the homogenous response of zebrafish to overfeeding and the rapid development of steatosis and fibrosis in this animal also make it a manageable tool for testing drugs or interventions for the treatment of NAFLD.

The aims of our study were therefore to assess, in a large cohort of individuals with histological diagnosis of NAFLD, whether menopausal status is associated with the severity of liver fibrosis, and to explore this same issue in an experimental model of liver steatosis in zebrafish.

## RESULTS

### Human NAFLD

#### Clinical features and histology

The baseline characteristics of both reproductive-age and menopausal women, as well as of their age-matched groups of males are reported in Table 1. Women at reproductive age, compared with age-matched males, had higher high-density lipoprotein (HDL) cholesterol serum levels (*P*<0.001), whereas no differences in other metabolic parameters and in histological severity of liver disease were observed. Similarly, in menopausal women, compared with age-matched males, there was a higher prevalence of obesity, as well as higher total (*P*<0.001) and HDL (*P*<0.001) cholesterol levels.

**Table 1.**
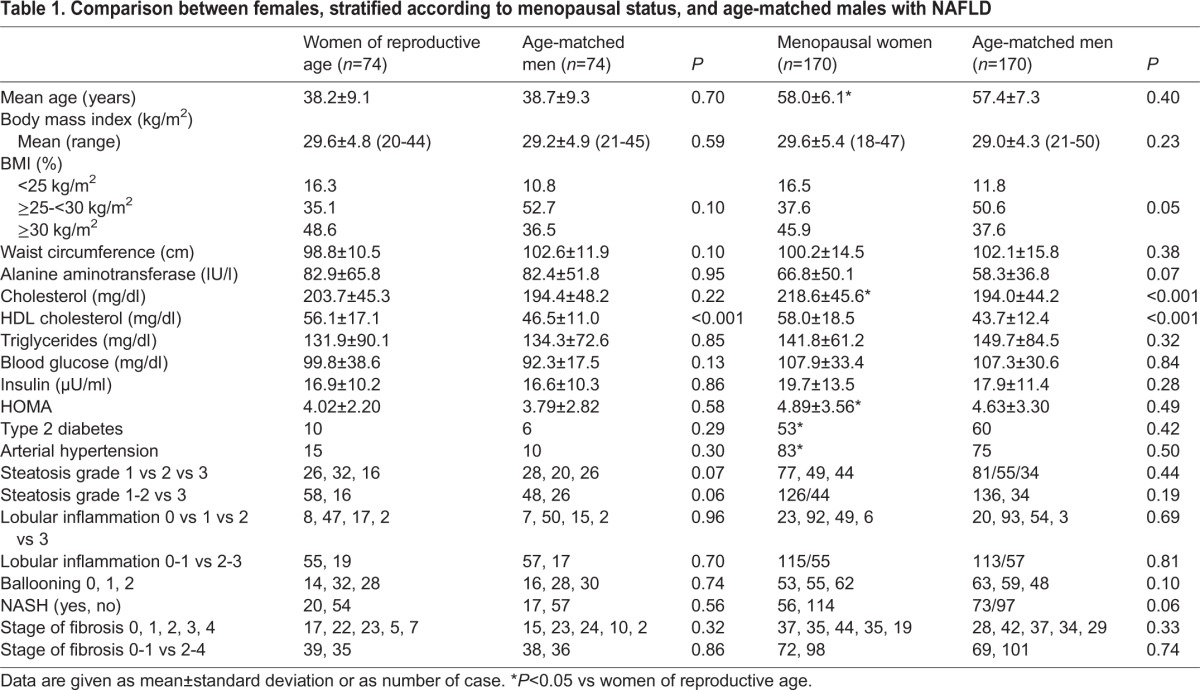
**Comparison between females, stratified according to menopausal status, and age-matched males with NAFLD**

When comparing reproductive age versus menopausal women, the latter had a higher prevalence of metabolic disturbances [higher total cholesterol, *P*=0.02; higher homeostasis model assessment (HOMA), *P*=0.05; type 2 diabetes, *P*=0.004; arterial hypertension, *P*<0.001], whereas no significant differences were present in the histological features.

Notably, 15 (9.6%) menopausal females were on HRT, all of them for a period equal to or longer than 6 months. No difference was observed in terms of metabolic variables by stratifying for HRT, but there was a lower prevalence of arterial hypertension (20% vs 51.6%, *P*=0.02) (supplementary material Table S1), whereas menopausal women on HRT compared with their counterparts not on HRT had a lower prevalence of NASH (33.3% vs 70.3%, *P*=0.009) and a trend towards a lower prevalence of F2-F4 fibrosis (33.3% vs 60%, *P*=0.08).

#### Factors associated with significant liver fibrosis (F2-F4)

Supplementary material Table S2 shows variables linked to F2-F4 fibrosis in the entire cohort of males and females. Specifically, by using women at reproductive age as reference, male gender [odds ratio (OR): 1.408, 95% confidence interval (95% CI): 0.779-2.542, *P*=0.25] was not independently associated with F2-F4 fibrosis, whereas a non-significant trend was observed for menopausal status in women (OR: 1.752, 95% CI: 0.956-3.208, *P*=0.06).

When considering females only, HDL (OR: 0.982, 95% CI: 0.964-1.000, *P*=0.04), menopause (OR: 2.717, 95% CI: 1.020-7.237, *P*=0.04), and NASH (OR: 3.214, 95% CI: 1.743-5.926, *P*<0.001) were independently linked to F2-F4 fibrosis (Table 2). To strengthen the concept that the effect of menopausal status in females was independent of age, we performed subgroup analyses by comparing women at different reproductive stages matched for age. Specifically, when comparing 20 fertile [mean age 48 years, mean body mass index (BMI) 30 kg/m^2^] with 20 age-matched menopausal (mean age 48 years, mean BMI 33 kg/m^2^) women, the latter had a significantly higher prevalence of F2-F4 fibrosis (six out of 20 vs 16 out of 20, *P*=0.001). When age was excluded from the model, menopause and F2-F4 fibrosis remained independently associated (OR: 2.934, 95% CI: 1.096-7.914, *P*=0.03).

**Table 2.**
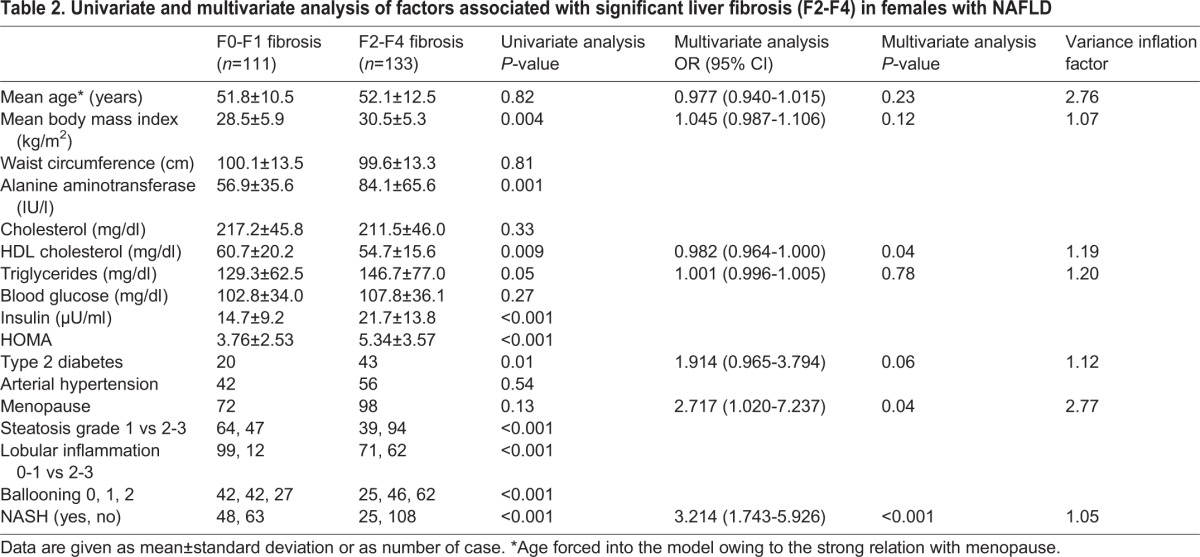
**Univariate and multivariate analysis of factors associated with significant liver fibrosis (F2-F4) in females with NAFLD**

Finally, when considering males only, the following variables were independently linked to F2-F4 fibrosis: age (OR: 1.039, 95% CI: 1.011-1.068, *P*=0.006), BMI (OR: 1.111, 95% CI: 1.029-1.200, *P*=0.007) and NASH (OR: 4.458, 95% CI: 2.405-8.264, *P*<0.001).

### Zebrafish NAFLD

#### Characterization of reproductive status

**Fig. 1.**
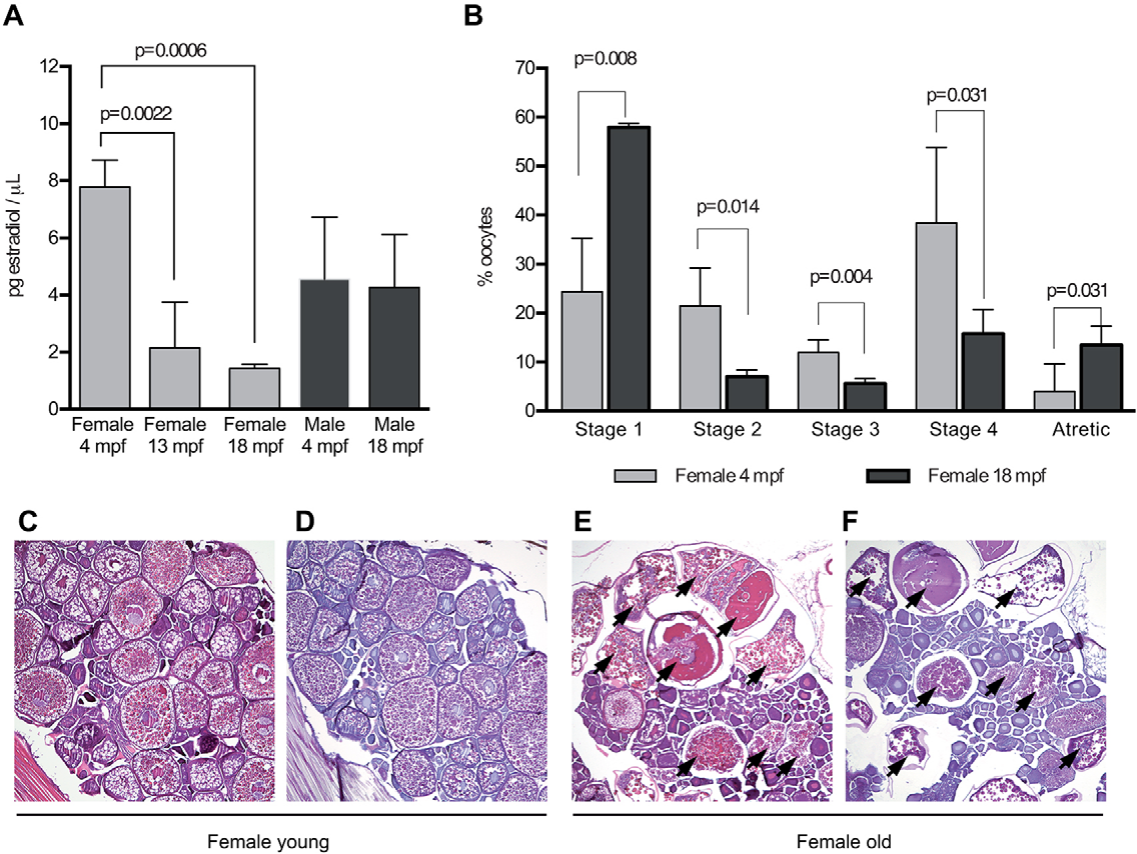
**Definition of reproductive age in zebrafish.** (A) Serum estradiol levels in female and male zebrafish during ageing. Data are reported as mean±s.d. Final sample number=12 per sex/age. (B) Application of the ovary maturation classification in the evaluation of the effects of ageing on zebrafish ovarian tissue (see supplementary material Fig. S1 for details). Three sections from five fish per subgroup were analyzed. Data were expressed as mean±s.d. H&E-stained (C,E) and PAS-stained (D,F) sections of the ovaries of young (4 mpf) and old (18 mpf) female zebrafish. Atretic follicles (indicated by arrows) were mostly found in old female fish with low to absent E2 levels. mpf, months post fertilization.

In order to study the effect of diet at different reproductive ages, we used young and old zebrafish. To substantiate the reproductive age, in particular the ovarian senescence in old female fish, we analyzed the circulating levels of estradiol and performed histopathologic evaluation of ovaries (Fig. 1).

As shown in Fig. 1A, circulating estradiol (E2) levels sharply decreased from fertile young female fish (4 months old) (7.7±0.94 pg/µl) to 18-month-old female fish (1.4±0.1 pg/µl) (*P*=0.0006), with intermediate values in 13-month-old fish (2.1±1.6 pg/µl) (4-month- vs 13-month-old fish *P*=0.0022) (Fig. 1A). E2 levels in both young and old male fish were significantly different from those values found in young and old female fish (young female vs young male fish: 7.7±0.94 pg/µl vs 4.5±2.15 pg/µl, *P*=0.0342; old female vs old male fish: 1.4±0.1 pg/µl vs 4.2±1.8 pg/µl, *P*=0.0013). E2 levels were not significantly different between young and old male fish.

Histopathological analysis revealed a significant difference between young and old females. Ovaries in young females showed a compact, tightly organized structure, with a high number of follicles in different stages of maturation (Fig. 1C,D). Ovaries in old female fish showed a much looser structure, with degeneration of ovarian tissue and presence of atretic follicles (Fig. 1E,F). Quantification of follicles at different stages of maturation (Fig. 1B; supplementary material Fig. S1) showed, in young female fish, a significantly higher number of mature follicles (i.e. stage 2 to 4) (young vs old female fish at stage 2, 3 and 4: *P*=0.014, 0.004, 0.031, respectively) in comparison with that in old female fish. Conversely, the percentages of stage 1 (*P*=0.008) and atretic follicles (*P*=0.031) were significantly higher in old females (Fig. 1B).

#### Body mass index

As a result of overfeeding, the BMI was already significantly increased after 3 weeks, in comparison with the baseline (*P*=0.04). A significant increase in BMI was evident from week 6 onwards in overfed zebrafish as compared with controls (*P*<0.05 at each time point) (supplementary material Fig. S2A). All overfed zebrafish (*n*=164) developed obesity after a median period of overfeeding of 16 weeks. Analysis of zebrafish BMI according to age and gender revealed that the median increase in BMI was 23% in old animals (both males and females), 26% in young males and 33% in young females (supplementary material Fig. S2B).

#### Histopathological evaluation of zebrafish liver

**Fig. 2.**
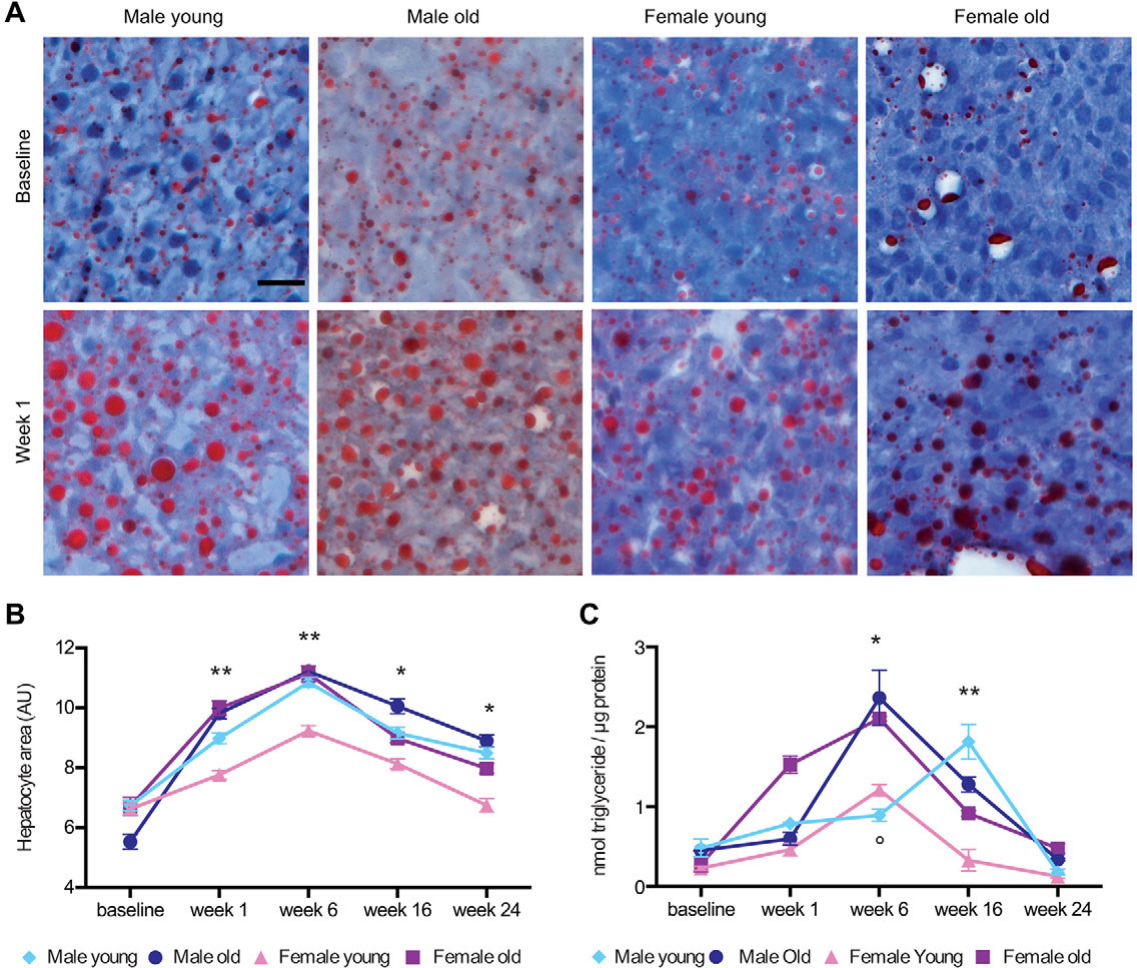
**Hepatic steatosis in overfed zebrafish.** (A) Oil Red O staining of liver sections from young males, old males, young females and old female zebrafish at baseline, and after 1 week of overfeeding. Scale bar: 20 µm. The percentage of the area stained by Oil Red O was quantified by using Image J (Schneider et al., 2012) in three sections from five fish per subgroups (supplementary material Fig. S3B). (B) To compare the development of steatosis between different groups, we measured, by using ImageJ (Schneider et al., 2012), the surface area of 30 randomly assigned hepatocytes in three sections from five different fish per subgroup, which had been stained with H&E (Kuroda et al., 2012). Values are expressed as means±s.e.m. The group of young females was compared with each of the other groups (*P<0.0001; **P<0.01). (C) Quantification of liver triglyceride in overfed zebrafish that had been grouped according to age and gender. Final sample number=6 per subgroup. Values are expressed as means±s.e.m. (*P<0.0001 young females vs old males and old females; °*P*=0.0049 young females vs young males; **P<0.0001, the group of young females was compared with each of the other groups).

Microscopic evaluation of haematoxylin and eosin staining (H&E) of control and overfed zebrafish liver tissue revealed the presence of hepatic steatosis in overfed zebrafish as early as 1 week after the beginning of the overfeeding protocol (supplementary material Fig. S3A). No changes were observed in the liver of control animals throughout the study. To further evaluate whether lipids accumulated in the liver, hepatic tissue was stained with Oil Red O. In overfed zebrafish, most of the cell cytoplasm was occupied by large fat vacuoles, as shown in Fig. 2A. A significant increase in Oil Red O staining was observed in all overfed groups but young females at week 1 compared with respective baseline fish (male young: *P*=0.033; male old: *P*=0.009; female young: *P*=0.283; female old: *P*=0.014) (supplementary material Fig. S3B). Periodic acid–Schiff staining (PAS) was used to identify the presence of glycogen. Control livers exhibited normal glycogen storage, whereas overfed livers revealed low levels of glycogen confined in the peripheral region of hepatocytes (supplementary material Fig. S3A).

A significant enlargement of hepatocyte area was observed: the increase in hepatocyte size was rapid, being already present in all age groups 1 week after the beginning of overfeeding (*P*<0.001 compared with the respective baseline for each subgroup). A significant increase in the area of the hepatocytes was observed in all four groups, although young female zebrafish, despite developing the largest BMI increase, had at all time points, significantly lower steatosis than the other three groups (Fig. 2B). Although hepatocyte area decreased after week 6, at week 24 it was still significantly higher than at baseline in male (both old and young) and old female groups (*P*<0.0001 for each intra-group and inter-group comparison) but not in young female, in which it decreased at week 24 to the level observed at week 1 (t_0_ vs t_24_: *P*=0.725) (Fig. 2B). Similar to steatosis, triglyceride levels in the liver significantly increased during the overfeeding period, reaching their maximum at week 6 in all considered groups but young males (male old and female old: *P*<0.0001 and female young: *P*=0.0002 vs their corresponding baseline). In young males, the highest triglyceride levels were observed at week 16 (week 16 vs baseline: *P*<0.001). Young females had significantly lower peak levels of liver triglyceride as compared with other groups (vs old males and old females at week 16: *P*<0.0001; vs young males at week 6: *P*=0.0092) (Fig. 2C).

**Fig. 3.**
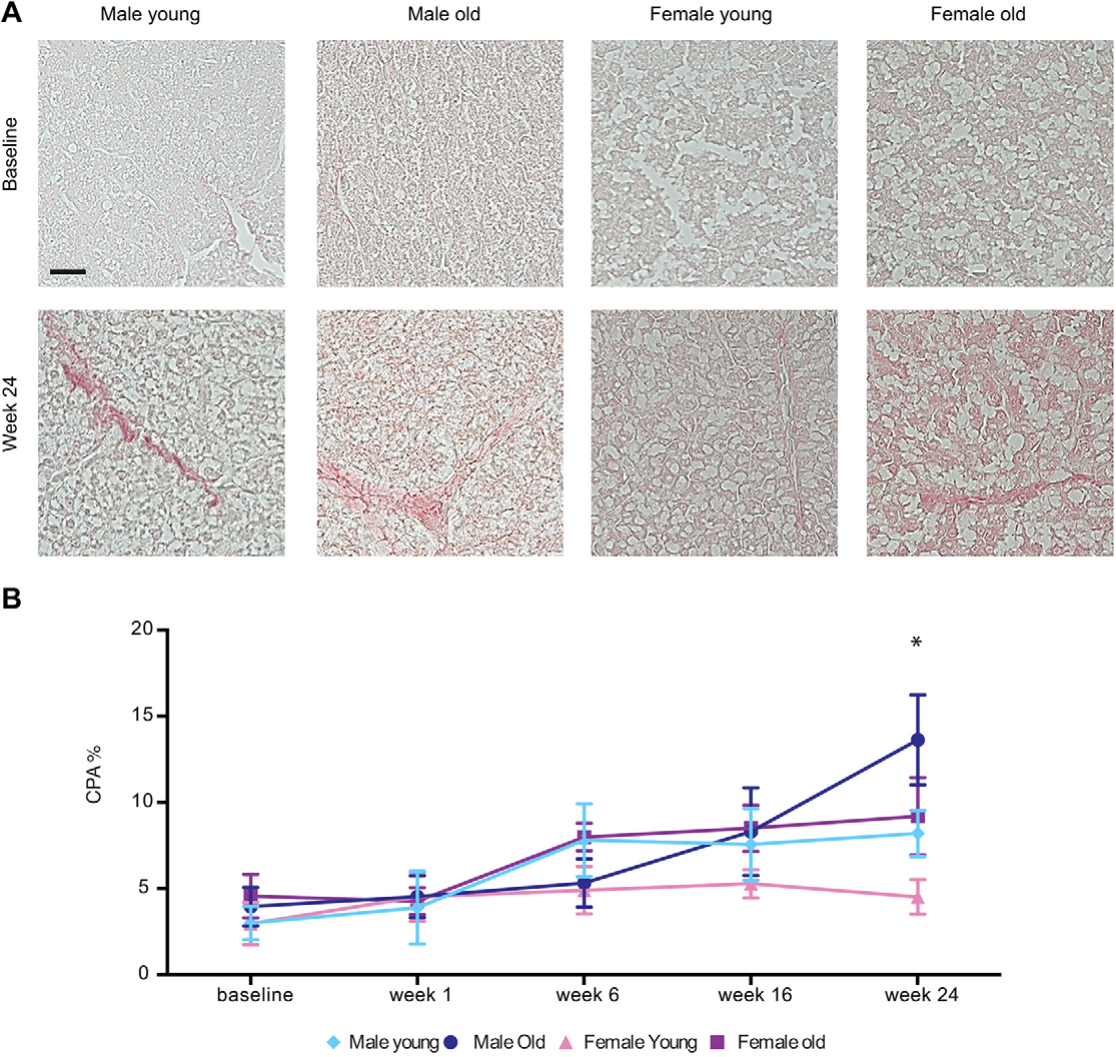
**Assessment of hepatic fibrosis in overfed zebrafish.** (A) Sirius Red staining of liver sections from young males, old males, young females and old female zebrafish at baseline, and after 24 weeks of overfeeding. Scale bar: 30 µm. (B) Quantification of liver fibrosis by using computer-assisted digital image analysis. Data are reported as collagen proportionate area (CPA) converted into a percentage. Three sections from five fish per subgroup were analyzed. Values are expressed as means±s.d. *P<0.0001 for young females versus all the other groups.

Liver fibrosis was assessed by using Sirius Red staining, which detects collagen (Fig. 3A). A significant increase in the level of fibrosis compared to that at baseline could be observed already at week 6 in young males (*P*<0.0001) and in old females (*P*=0.038). Fibrosis further increased at week 16 (young males: *P*<0.0001; old males: *P*=0.0003; old females: *P*=0.011 all vs their baseline) and at week 24 (young males: *P*<0.0001; old males: *P*<0.0001; old females: *P*=0.004 all vs their respective baseline). At all time points analyzed, young females did not show any increase in fibrosis as compared with their basal level (Fig. 3B). At week 24, young females had a significantly lower level of fibrosis as compared with that of all other groups (*P*<0.001 vs young males, old males and old females).

#### Deregulation of genes involved in lipid metabolism and liver damage

**Fig. 4.**
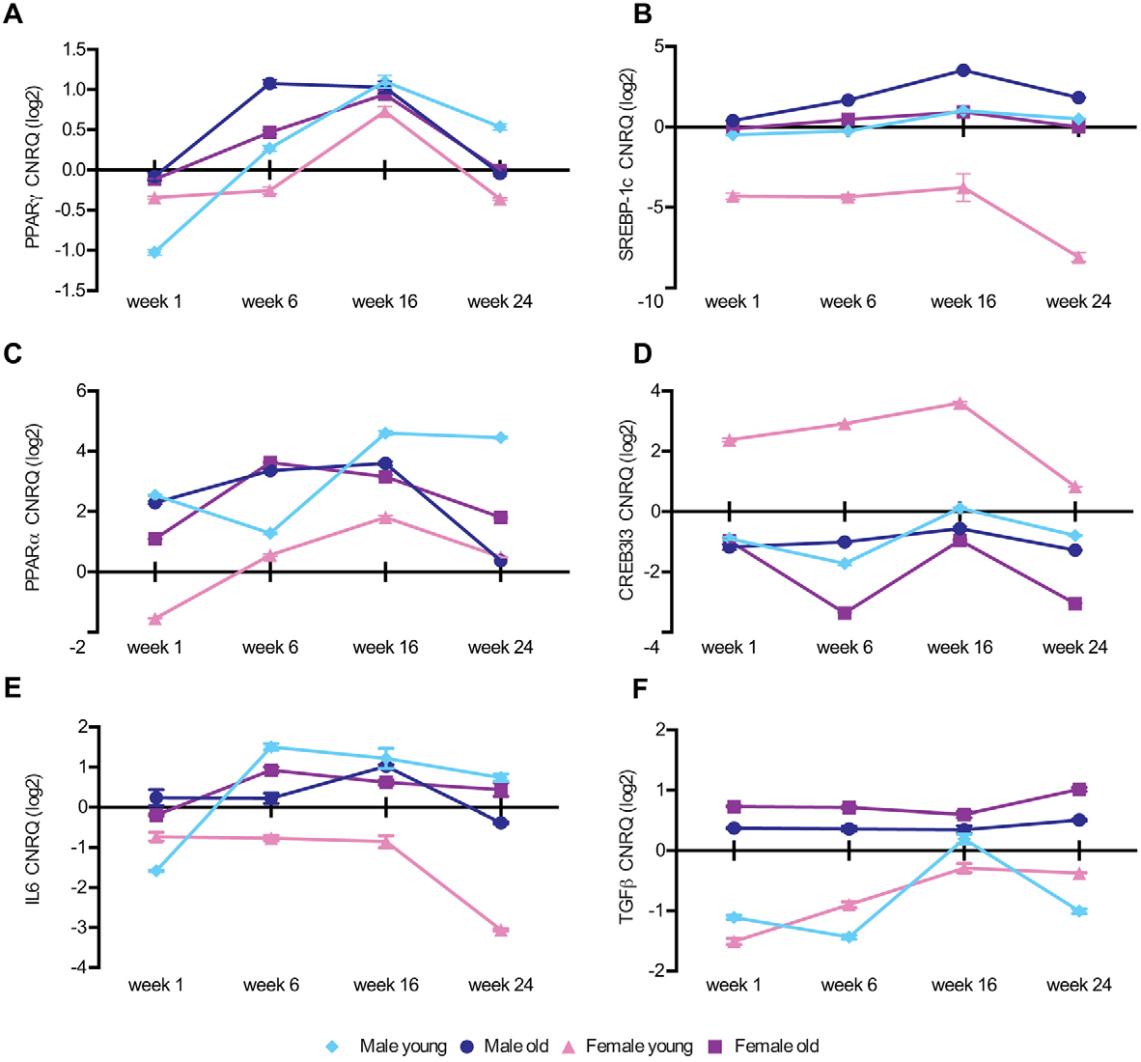
**RT-qPCR analysis for the expression of marker genes in the livers of overfed zebrafish.** Genes involved in lipid metabolism (A-D), inflammation (E) and fibrosis (F) in zebrafish. Samples from five fish per subgroup were analyzed. Gene expression was reported as the calibrated normalized relative quantity (CNRQ) geometric mean±s.e.m. of each group expressed as log(2). Each value was normalized to the CNRQ geometric mean of the corresponding control.

To investigate the mechanisms underlying the development and progression of liver damage in obesity-associated conditions, we analyzed the expression of marker genes involved in lipid metabolism (*pparg*, *ppara* and *sreb1c*), hepatic lipogenesis and fatty acid oxidation (*creb3l3*), inflammation (*il6*) and fibrosis (*tgfb*) (Fig. 4). Quantitative real-time PCR (RT-qPCR) analyses showed increased transcript levels for *pparg* and *srebp1c*, and decreased *creb3l3* transcript levels at weeks 6 and 16 in all groups but young females, in which the reverse occurred (Fig. 4A,B,D). Upregulation of *ppara* was observed in all groups as compared with controls. However, in young females, the levels of *ppara* mRNA were significantly lower as compared with that of all the other groups (Fig. 4C). Increased expression of the *il6* transcript was found in males and old females from week 6, and in old males at week 16. In young females, *il6* was downregulated at all time points (Fig. 4E). *tgfb* showed an increased expression in older animals from week 1, as compared with basal levels. In young animals (both males and females), *tgfb* expression was mostly downregulated (Fig. 4F).

## DISCUSSION

In this study, which considered a large cohort of females and age-matched males by using histological diagnosis of NAFLD, we found that menopause is associated with a significantly higher risk of relevant liver fibrosis (i.e. equal to or higher than F2). By contrast, women at reproductive age are at significantly lower risk of developing relevant liver fibrosis. The same negative effect of ovarian senescence and the protective effect of fertile age were also found in female zebrafish with experimental steatosis.

Epidemiological studies report a higher prevalence of NAFLD in males compared with females, and such studies also indicate an increase in NAFLD prevalence and incidence in menopausal females in comparison with reproductive-age females (Chow et al., 2011; Hewitt et al., 2004; Itagaki et al., 2005; Yasuda et al., 1999). However, data on the impact of gender and of menopausal status on liver disease severity are few and inconclusive. We observed that in the entire cohort (males and females), childbearing age was associated with a trend towards a lower prevalence of significant (stage F2-F4) liver fibrosis, this effect being observed in comparisons with menopausal females but not with the entire cohort of males. Of note, this association between menopause and increased fibrosis was maintained after adjustment for age, a strong determinant of menopause and fibrosis progression; for metabolic comorbidities associated with menopause, such as obesity and diabetes; and for histological features of NASH. Taken together, our data strongly add to the understanding of the effect of menopause on liver fibrosis in NAFLD. A recent study from the USA (Yang et al., 2014) on a cohort of 541 consecutive subjects (352 females) with biopsy-proven NASH has also reported the protective effect of being of reproductive age on fibrosis. Because two different cohorts, one from Europe and one from the USA, gave similar results, a meta-analytic pooling of the two cohorts would be useful to further explore the clinical impact of menopause on liver damage in NAFLD and to identify clinical subgroups at higher risk.

The more relevant clinical finding of our study lies in the fact that, for the first time to our knowledge, we assessed the impact of menopausal status on the severity of liver damage in females with NAFLD. We clearly showed that menopause is associated with a higher risk of significant liver fibrosis, which is independent of age and of metabolic comorbidities. The strong association between menopause and liver fibrosis, possibly related to the much stronger surge of inflammatory activation associated with menopause, is in line with data in chronic hepatitis C (Villa et al., 2011, 2012). These data, if confirmed, could prompt a more careful management of NAFLD pathogenic factors during the first phases of menopause to reduce the progression of liver damage, also suggesting that there is an opportunity to use HRT to delay liver damage progression. In line with this, similar to other studies (McKenzie et al., 2006), we reported lower levels of liver damage in menopausal females with NAFLD that were undergoing HRT therapy compared with those that were not.

Our clinical evidence is further supported by data obtained in a model of overfed zebrafish*.* The first advantage of this model, in comparison with mice, is the very high occurrence of obesity and steatosis (Buettner et al., 2007; Burcelin et al., 2002). Indeed, all overfed fish developed obesity and fatty liver. Aside from this high occurrence, the zebrafish model has another relevant advantage over mice, with respect to the evaluation of fibrosis development according to reproductive stages – the occurrence of ovarian senescence in zebrafish is spontaneous, whereas in mice it has to be induced through an ovariectomy (Gopalakrishnan et al., 2013). The 18-month-old and older female zebrafish is a convincing ‘menopausal prototype’ because the very low circulating E2 levels and ovarian histology (Fig. 1; supplementary material Fig. S1) are indicators of the occurrence of spontaneous ovarian senescence. E2 levels were significantly lower in old versus young female zebrafish (*P*=0.0006) and lower, but to a lesser extent, in 13-month-old zebrafish (*P*=0.048), indicative of a progressive hormonal decline similar to that observed in women when transitioning from reproductive age to menopause (Villa et al., 2012). Furthermore, in old versus young zebrafish, a significantly higher number of immature and atretic follicles, characteristic of ovarian senescence, were present. By contrast, in young fertile zebrafish, the number of maturing follicles was significantly higher than that in older zebrafish (Fig. 1; supplementary material Fig. S1). Female zebrafish at 18 months of age and older can therefore be convincingly considered to be in a menopause-like state.

With our model, we showed that old overfed female zebrafish developed hepatic steatosis and fibrosis in a manner similar to overfed males (both young and old). By contrast, young female fish, despite a high increase in BMI, developed less steatosis and were completely protected from the development of fibrosis. Gene expression analysis supports the view that simple overfeeding is able to start a series of events that lead first to liver steatosis and then to fibrosis, and that the ovarian senescence increases the risk of fibrosis. Old overfed female zebrafish exhibited a pattern of gene expression in the liver that is associated with hepatic fat accumulation, similar to males. Specifically, females showed an increase in *pparg* and *srebp1c,* two genes that are known to be involved in activation and *de novo* lipogenesis, as well as the hepatic synthesis of fatty acid (Hsu and Huang, 2006; Tailleux et al., 2012; Yu et al., 2003), and a decrease in *creb3l3*, which has a protective role with regards to the development of steatosis (Zhang et al., 2012). Upregulation of *tgfb* expression in old animals (both males and females), and of note of *il6* expression in old animals and young males (i.e. those showing the highest levels of fibrosis both through histology and digital imaging evaluation) also supports our hypothesis (Bayer et al., 1998; Collins et al., 2013; Park et al., 2010). The data in zebrafish, together with the known lower expression of estradiol receptor in the liver of individuals with NASH (Erkan et al., 2013) and the inhibitory effect of estradiol on expression of *TGFB1*, *TNFA* (Itagaki et al., 2005; Yasuda et al., 1999), *IL6* and *IL1B* (Kireev et al., 2010; Rogers and Eastell, 2001) also suggest a protective effect of fertile status on the severity of liver damage in NAFLD. The marked upregulation of hepatic *TNFA* and *IL6* at the time of early menopause, becoming non-significant in late menopause, could explain the strong but selective association we observed between liver fibrosis and early menopause.

A limit of our study lies in the lack of data, among the NAFLD population, on serum estrogen levels that, instead of menopausal status, could give a direct measure of the link between ovarian reserve and both histological and metabolic disturbances, as already observed for liver fibrosis in chronic hepatitis C (Villa et al., 2012), and for cholesterol levels in other clinical settings (Persson et al., 2012; Ramezani Tehrani et al., 2014).

In conclusion, this study on a large cohort of women and age-matched men with histological diagnosis of NAFLD shows that menopausal status increases the risk of fibrosis severity. These data were supported and reinforced by our findings in overfed zebrafish with experimental steatosis. These results, which need validation in other NAFLD settings, suggest that a more intensive management should be planned for females with NAFLD in pre- and early menopause.

## MATERIALS AND METHODS

### Subjects

We analyzed data from 244 consecutive Italian women (meaning that they presented within a certain time period and that none were excluded) prospectively recruited at five referral centers from Palermo (*n*=86), Turin (*n*=67), Milan (*n*=39), Rome (*n*=34) and Modena (*n*=18), and 244 center age-matched males, all with a clinical and histological diagnosis of NAFLD. Individuals with other causes of liver disease (alcoholic, viral, autoimmune, hereditary hemochromatosis and α1-antitrypsin deﬁciency), with advanced cirrhosis and/or hepatocellular carcinoma, and with current use of steatosis-inducing drugs were excluded.

The study was performed in accordance with the principles of the Helsinki Declaration, and with local and national laws. Approval was obtained from the hospital Internal Review Boards and the Ethics Committees (124/11 Men_Epid, prot.n.2420/CE), and informed consent for the study was obtained from all controls and test subjects.

#### Clinical and laboratory assessment in human NAFLD

Clinical and anthropometric data were collected at the time of liver biopsy. BMI was calculated on the basis of weight in kilograms and height in meters. The diagnosis of arterial hypertension was based on the following criteria – systolic blood pressure ≥135 mmHg and/or diastolic blood pressure ≥85 mmHg (measured three times within 30 min, in the sitting position and using a brachial sphygmomanometer), or use of blood-pressure-lowering agents. The diagnosis of type 2 diabetes was based on the revised criteria of the American Diabetes Association, using a value of fasting blood glucose ≥126 mg/dl on at least two occasions (American Diabetes Association, 2000). In individuals with a previous diagnosis of type 2 diabetes, current therapy with insulin or oral hypoglycemic agents was documented. Menopause was defined as no menstrual periods for 12 consecutive months; use of HRT was recorded.

A 12-h overnight fasting blood sample was drawn at the time of biopsy to determine serum alanine aminotransferase, total cholesterol, HDL cholesterol, triglycerides, and plasma glucose and insulin concentrations. Insulin resistance was assessed by using HOMA with following equation (Matthews et al., 1985) – insulin resistance (HOMA−IR)=fasting insulin (μU/ml)×fasting glucose (mmol/l)/22.5 (Ikeda et al., 2001).

#### Assessment of histology in human NAFLD

Slides were coded and read at each clinical center by one expert pathologist, who was unaware of the identity and history of individuals. A minimum 15-mm length of the biopsy specimen or the presence of at least 10 complete portal tracts was required (Colloredo et al., 2003). Steatosis was assessed as the percentage of hepatocytes containing fat droplets (minimum 5%) and evaluated as a continuous variable. Kleiner classification (Kleiner et al., 2005) was used to compute steatosis, ballooning and lobular inflammation, and to stage fibrosis from 0 to 4. NASH was considered to be present when steatosis, ballooning and lobular inﬂammation were present.

### Animals

Adult zebrafish were supplied by the Zebrafish International Research Centre (Eugene, OR, USA), or bred in-house. The average age for the ‘young’ zebrafish group was 4 months, whereas that for the ‘old’ groups was 18 months (Shao et al., 2011). Animals were maintained in a ZebTec system (Tecniplast, Buguggiate, Varese, Italy) at 28°C, 14 h:10 h light:dark cycle at a density of 3-6 fish/l (Castranova et al., 2011). This density was chosen as it does not affect fertility, size or viability of fish. All efforts were made to minimize animal suffering and to reduce the number of animals used, in accordance with the European Communities Council (2010/63/UE) and with the requirements of the veterinary institutional review board of the University of Modena and Reggio Emilia (2007/06_22).

#### Characterization of reproductive status

##### Determination of circulating estradiol levels

Zebrafish were anesthetized in ice-cold water, and blood was drawn from an incision just between the anal fin and the caudal fin with a heparinized pipette tip. Blood from two fish of the same sex and age was pooled as one replicate (final sample number=12 per sex/age).

Serum was diluted with 200 μl double-distilled water, and the concentration of estradiol was determined with EIA Estradiol Kit (Cayman Chemical, Ann Arbor, MI, USA) following the manufacturer's protocol.

##### Histopathological analysis of ovaries

To substantiate reproductive status, young and old female fish at enrolment were euthanized and fixed by immersion for 24 h in neutral buffered 10% formalin and paraffin-processed. Paraffin-embedded samples were cut into a 5-μm-thick section and stained by H&E and PAS staining.

Eggs are evidenced in ovaries in four maturation stages – stage 1 (primary growth stage), stage 2 (cortical alvelolus stage), stage 3 (vitellogenic stage) and stage 4 (mature phase). Menopausal ovaries are characterized by atretic stage – the vitelline membrane structure starts to disintegrate, the vitelline envelope breaks down and yolk reabsorption takes place (supplementary material Fig. S1) (Aytekin and Yüce, 2008; Marlow and Mullins, 2008).

#### Feeding zebrafish

Zebrafish were separated into two dietary groups (overfeeding and control groups). Within the two dietary groups, zebrafish were divided into four groups according to gender and age. Animals in the overfeeding group were fed three times a day with *artemia nauplii* (11.6% lipid, 20.0% carbohydrate, 61.6% protein, 6.8% ash; about 5013 cal g^−1^) (Zebrafish Management, Winchester, UK) with 20 mg/fish, whereas fish in the control group were fed twice a day with 5 mg/fish (Agh et al., 2008; Oka et al., 2010). Overweight was defined as a 10% BMI increase, and obesity as a 20% increase in comparison with baseline BMI.

#### Histopathological analysis of zebrafish

Zebrafish were euthanized after anesthesia with 0.1 mg/ml tricaine (Sigma-Aldrich S.R.L., Milan, Italy) (baseline and after 1, 6, 16 and 24 weeks of diet), and cryo-embedded in OCT (Miles Inc., Elkhart, USA) or fixed by immersion for 24 h in neutral buffered 10% formalin and paraffin-processed. Frozen 5-μm cryostat sections were stained with 0.5% Oil Red O (BDH Laboratory Supplies, Dorset, UK) in propylene glycol and counterstained with Carazzi's haematoxylin (Sigma-Aldrich S.R.L., Milan, Italy).

Paraffin-embedded samples were cut into 5 μm-thick sections and stained by using H&E, Sirius Red or PAS staining.

0.1% Sirius Red (Chroma-Gesellschaft, Munster, Germany) in pyric acid was applied for 50 min without haematoxylin counterstaining. Samples were observed by using light microscopy Nikon Eclipse 80i (Nikon Instruments Spa, Campi Bisenzio, Florence, Italy) and acquired with a Nikon Ds-Fi2 digital color camera (Nikon Instruments Spa, Campi Bisenzio, Florence, Italy).

#### Quantification of liver steatosis and fibrosis in zebrafish

Liver steatosis was quantified by measuring the area of hepatocytes using the National Institutes of Health ImageJ software (http://rsbweb.nih.gov/) (Kuroda et al., 2012; Schneider et al., 2012). Liver fibrosis was evaluated by using computer-assisted digital image analysis of histological sections stained with Sirius Red; hepatic collagen content was expressed as collagen proportionate area (CPA) converted into a percentage (Calvaruso et al., 2009). The collagen proportionate area was measured by using Zeiss Axiovision image analysis software (rel 4.8) (Zeiss, Germany).

#### Triglyceride quantification in Zebrafish

Triacylglycerol levels in the liver were determined using Triglyceride Quantification Kit (Biovision, Milpitas, CA, USA), according to manufacturer's protocol. Triglyceride levels were normalized to the protein level in the liver. The protein levels in the liver were determined using a colorimetric Protein Quantitation Kit (Euroclone, Milan, Italy) according to manufacturer's protocol.

#### RNA extraction and RT-qPCR in zebrafish

Total RNA was extracted from liver samples that had been frozen in liquid nitrogen by using Nucleo Spin miRNA kit (Macherey-Nagel, Duren, Germany) following the manufacturer's protocol. cDNA synthesis was performed using iScript Reverse Transcription Supermix (Bio-Rad Laboratories, Hercules, CA, USA). RT-qPCR was performed on cDNA samples using LightCycler 480 SYBR Green I Master Mix (Roche Diagnostics, Indianapolis, IN, USA) and a Light Cycler 480 Real Time PCR system (Roche Diagnostics, Indianapolis, IN, USA). The primer-sets can be found in supplementary material Table S3. The mRNA expression was normalized to that of *gapdh*, *ef1a* and *actb* mRNA using qBASEplus 2.4 (Hellemans et al., 2007). Data were normalized on the corresponding control and reported as log(2) mean±s.e.m. of biological replicates.

#### Statistics

Continuous variables were summarized as mean±standard deviation, and categorical variables as frequency and percentage. The Student's *t*-test and the chi-square test, as well as ANOVA and Holm-Sidak tests were used when appropriate (GraphPad Prism Software, San Diego, CA, USA). Multiple logistic regression models were used to assess the factors that were independently associated with significant fibrosis in the entire cohort, and in males and females considered separately. In the models, the dependent variable was fibrosis, coded as 0=not significant (F0-F1), and 1=significant (F2-F4). Variables associated with the dependent variable on simple logistic regression (probability threshold, *P*<0.10) were included in multiple logistic regression. Age and menopause were also included in the models if *P*>0.10 on simple regression because of their biological plausibility. To avoid the effect of co-linearity, HOMA score, blood glucose, insulin levels and diabetes, as well as waist circumference and BMI, or total and HDL cholesterol, or NASH and steatosis, ballooning and lobular inflammation, were not included in the same multivariate model. Regression analysis was performed using PROC LOGISTIC, PROC REG, and subroutines in SAS (SAS Institute Inc., 1992).

## Supplementary Material

Supplementary Material
