# Supplementary material for: Ovarian senescence increases liver fibrosis in humans and zebrafish with steatosis
Source: Dis Model Mech. 2015 Sep 1;8(9):1037–46. doi: 10.1242/dmm.019950 (PMC4582103; doi:10.1242/dmm.019950)
Supplement: Supplementary Material [file supp_8_9_1037__index.html]

Supplementary Material 

# Ovarian senescence increases liver fibrosis in humans and zebrafish with steatosis

## DMM019950 Supplementary Material

- Supplementary Material
