# Supplementary material for: Ovarian senescence increases liver fibrosis in humans and zebrafish with steatosis
Source: Dis Model Mech. 2015 Sep 1;8(9):1037–46. doi: 10.1242/dmm.019950 (PMC4582103; doi:10.1242/dmm.019950)
Supplement: Supplementary Material [file supp_019950_DMM019950supp.pdf]

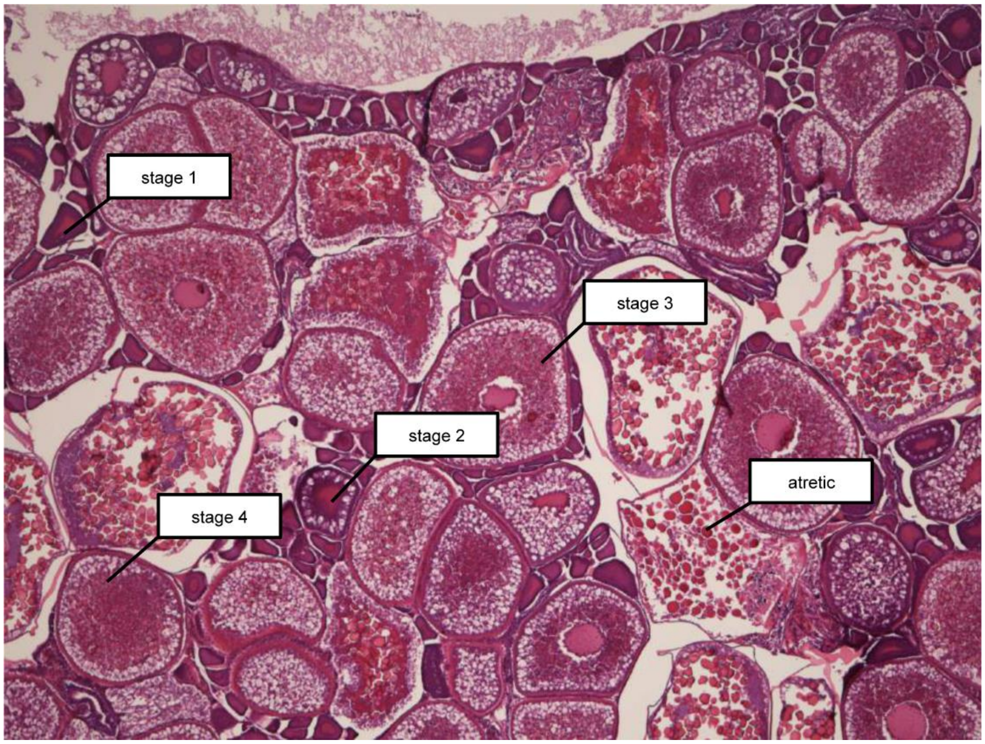

Supplemental figure 1. Developmental stages of the oocytes. Stage 1, perinucleolar oocytes. The oocytes size was small and ooplasm lacks granular structure. Stage 2, cortical alveolar oocytes. The vitelline envelope (zona radiata) begin to form. Stage 3, vitellogenic oocyte. The oocyte increased in size; the granular structures in ooplasm were larger and the nucleus was irregular in the shape. Stage 4, mature oocyte. The nucleus could not be observed due to the granular structures that filled up the entire cytoplas. Atretic stage. Oocyte and the vitelline membrane structure started to disintegrate.

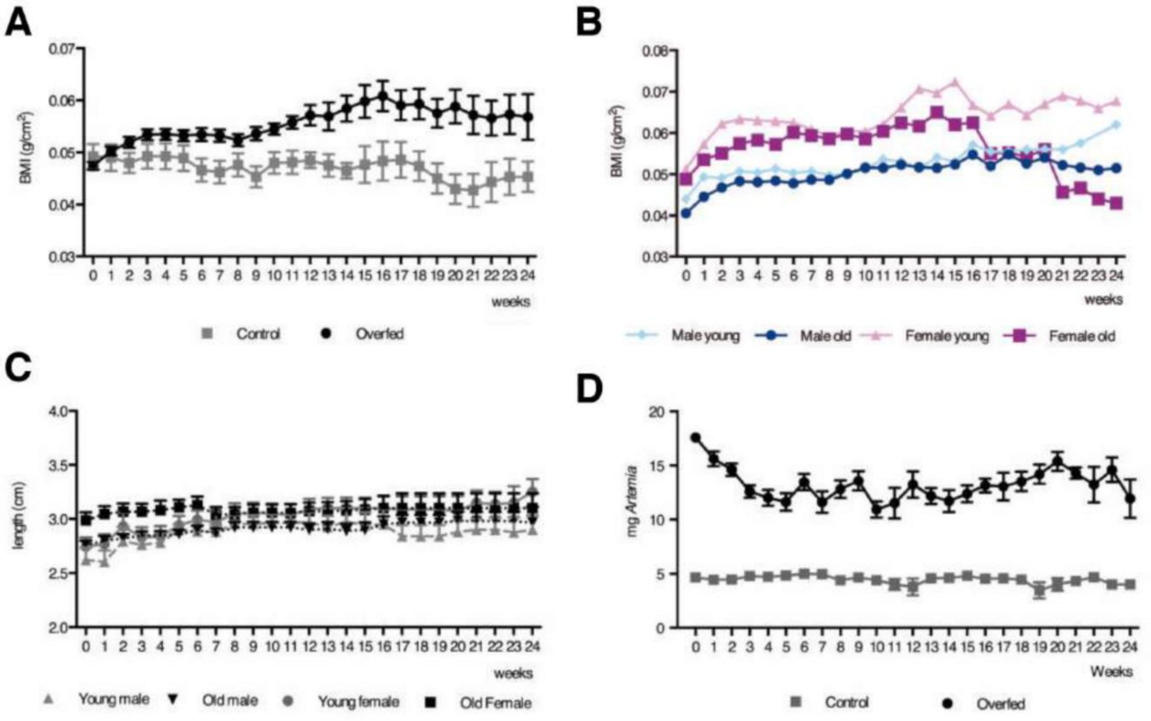

Supplemental figure 2. Evaluation of BMI, length and food consumption in Zebrafish overfed with *Artemia nauplii.* (A) BMI (g/cm2) in overfed zebrafish (n=164) compared with control zebrafish (n=59). Values are expressed as means ± SEM. BMI constantly increased during the first 16 weeks of the dietary protocol reaching its maximum at week 16. From week 17 to 24 BMI reached a plateau. (B) Mean BMI in overfed zebrafish grouped according to age and gender (young males n=39, old males n=43, young females n=39 and old females=43). BMI strongly increased in the first weeks of overfeeding but it was almost stable after week 16 in all groups but old females. In this group BMI started to decrease after week 16 reaching, by the end of the observation period, a much lower BMI than at baseline although no relevant change was observed throughout the study in the length of animals (C). (C) Length in cm of overfed zebrafish grouped accordingly with age and gender. Data were expressed as mean ± SEM (B) The percentage of eaten *Artemia* was measured by counting the number of *Artemia* in the feeding tank, before and after feeding as previously described (Oka et al.2010) and calculated on the total amount of given *Artemia.*

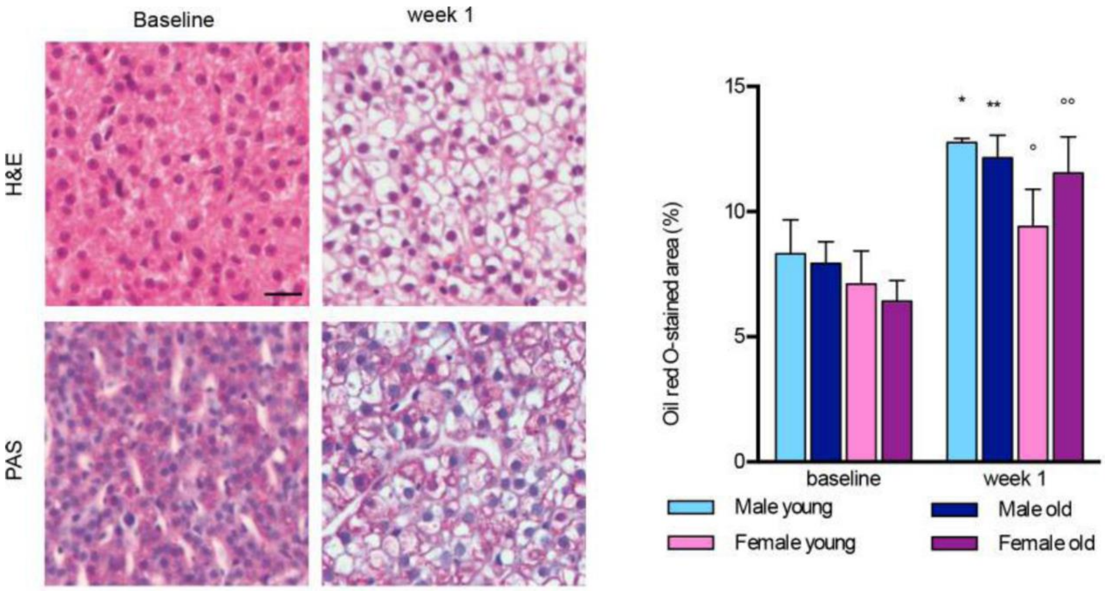

Supplemental figure 3. Hepatic steatosis in overfed zebrafish. (A) Representative H&E, and PAS staining of liver sections from controls (left) and overfed fish after 1 week of treatment (right). Both controls and treated animals were old male fish. Size bar 20 mm. (B) Quantitation of Oil red O stained area in liver sections from young males, old males, young females and old females zebrafish at baseline and after 1 week of overfeeding. The positive areas were measured with Image J (Schneider et al., 2012) and normalized on the total area of the section. The analysis has been carried out in three sections from 5 fish per subgroups. (* male young: p=0.033; ** male old: p=0.009; ° female young: p=0.283; °° female old: p=0.014; vs. respective baseline).

Supplemental Table 1. Characteristics of menopausal females with non-alcoholic fatty liver disease according to hormonal replacement therapy.

	Menopausal Women No HRT (n=155)	Menopausal Women on HRT (n=15)	P value
**Mean Age – yrs**	57.9 ± 6.2	58.5 ± 5.6	0.73
**Mean Body Mass Index – Kg/m^2**	29.7 ± 5.6	28.6 ± 3.9	0.49
**Alanine Aminotransferase – IU/L)**	66.4 ± 49.3	72.2 ± 59.2	0.67
**Cholesterol – mg/Dl**	217.8 ± 45.0	227.8 ± 52.4	0.43
**HDL Cholesterol – mg/Dl**	58.4 ±19.0	54.0 ±10.6	0.40
**Triglycerides – mg/Dl**	139.7 ± 61.2	165.0 ± 57.4	0.13
**Blood Glucose – mg/Dl**	108.4 ± 34.2	102.7 ± 23.1	0.54
**Insulin – µU/Ml**	19.5 ± 13.7	17.4 ± 7.7	0.63
**HOMA**	4.96 ± 3.67	4.05 ± 1.84	0.37
**Type 2 Diabetes**	50	3	0.41
**Arterial Hypertension**	80	3	0.02
**Steatosis Grade 1 vs. 2/3**	69/86	8/7	0.71
**Lobular Inflammation 0/1 vs. 2/3**	103/52	12/3	0.36
**NASH**	109	5	0.009
**F2-F4 Fibrosis**	93	5	0.08

Data are given as mean ± standard deviation or as number of case.

Supplemental Table 2. Univariate and multivariate analysis of factors associated with significant liver fibrosis (F2-F4) in the entire cohort of female and age-matched male patients with non-alcoholic fatty liver disease.

	F0-F1 Fibrosis (n=218)	F2-F4 Fibrosis (n=270)	Univariate Analysis (*P*)	Multivariate Analysis OR (95% CI)	Multivariate Analysis (*P*)
**Male gender**	107	137	.71		
**Mean Age – yrs**	50.8 ± 10.8	52.7 ± 12.3	.07	1.018 (0.998-1.039)	.07
**Mean Body Mass Index – Kg/m^2**	28.1 ± 4.4	30.3 ± 5.0	<.001	1.069 (1.022-1.119)	.004
**Waist Circumference- cm**	99.0± 11.5	102.6± 15.5	.02		
**Alanine Aminotransferase – IU/L)**	60.4 ± 37.7	75.3 ± 57.1	.001		
**Cholesterol – mg/Dl**	210.0 ± 44.4	199.3 ± 48.0	.01		
**HDL Cholesterol – mg/Dl**	54.0 ±18.0	48.5 ±15.0	<.001	0.980 (0.966-0.994)	.004
**Triglycerides – mg/Dl**	134.7 ± 67.2	147.7 ± 82.7	.06	1.000 (0.997-1.003)	.87
**Blood Glucose – mg/Dl**	100.1 ± 28.5	107.3 ± 34.0	.01		
**Insulin – μU/Ml**	13.8 ± 7.9	21.5 ± 13.2	<.001		
**HOMA**	3.55 ± 2.25	5.26 ± 3.63	<.001		
**Type 2 Diabetes**	38	91	<.001	1.833 (1.111-3.024)	.01
**Arterial Hypertension**	73	110	.14		
**Male Gender/Menopause**	179/39	235/35	.13	1.559 (0.902-2.694)	.11
**Male Gender** vs. fertile women				1.408 (0.779-2.542)	.25
**Menopause** vs. fertile women				1.752 (0.956-3.208)	.06

**Steatosis Grade 1 vs. 2/3**	110/108	102/168	.005		
**Lobular Inflammation 0/1 vs. 2/3**	186/32	154/116	<.001		
**Ballooning 0/1/2**	94/71/53	52/104/114	<.001		
**NASH**	109/109	57/213	<.001	3.799 (2.477-5.826)	<.001

Data are given as mean ± standard deviation or as number of case.

Supplemental Table 3 - Primers used for zebrafish qRT-PCR. Creb3l3 primers were designed in our laboratory by means of Primer3.

Gene	Forward Primer	Reverse Primer	Reference
pparg	5'- CCTGTCCGGGAAGACCAGCG-3'	5'-GTGCTCGTGGAGCGGCATGT-3'	Her et al.2011
srebp1c	5'-CAGAGGGTGGGCATGCTGGC-3'	5'-ATGTGACGGTGGTGCCGCTG-3'	Her et al. 2011
ppara	5'-CTGCGGGACATCTCTCAGTC-3'	5'-ACCGTAAACACCTGACGACG-3'	Her et al.2011
creb3l3	5'-GCCACTCTGTCCGAATCTCA-3'	5'-TGACTGAGGTGGGTTTCTGC-3'	
il6	5'-TCAACTTCTCCAGCGTGATG-3'	5'-TCTTTCCCTCTTTTCCTCCTG-3'	Varela et al. 2012
tgfb	5'-GCACGGATAAGTTCCTCTTCAC-3'	5'-CGAAAGTCAATGTAAAGCTTGC-3'	Liu et al. 2012
gapdh	5'-TTCTCACAAACGAGGACACAA-3'	5'-CAAGGTCAATGAATGGGTCA-3'	Oka et al.2010
ef1a	5'-GTGCTGTGCTGATTGTTGCT-3'	5'-TGTATGCGCTGACTTCCTTG-3'	Choi et al.2010
actb	5'-ATTGCTGACAGGATGCAGAAG-3'	5'-GATGGTCCAGACTCATCGTACTC-3'	Chu et al. 2012
